# Elevation of MMP-9 Levels Promotes Epileptogenesis After Traumatic Brain Injury

**DOI:** 10.1007/s12035-018-1061-5

**Published:** 2018-04-17

**Authors:** Barbara Pijet, Marzena Stefaniuk, Agnieszka Kostrzewska-Ksiezyk, Photini-Effie Tsilibary, Athina Tzinia, Leszek Kaczmarek

**Affiliations:** 1 Laboratory of Neurobiology, Department of Molecular and Cellular Neurobiology, Nencki Institute of Experimental Biology, Pasteura 3, 02-093 Warsaw, Poland; 2 Department of Neuroscience, University of Minnesota, Minneapolis, MN 55405 USA; 3 Brain Sciences Center, Minneapolis, MN 55417 USA; 4 Laboratory of Cell and Matrix Pathobiology, Institute of Bioscience and Applications, NCSR Demokritos, 153 10 Aghia Paraskevi Attikis, Athens, Greece

**Keywords:** Traumatic brain injury, Controlled cortical impact, Matrix metalloproteinase-9 (MMP-9), Extracellular matrix (ECM), Epileptogenesis, Posttraumatic epilepsy

## Abstract

Posttraumatic epilepsy (PTE) is a recurrent seizure disorder that often develops secondary to traumatic brain injury (TBI) that is caused by an external mechanical force. Recent evidence shows that the brain extracellular matrix plays a major role in the remodeling of neuronal connections after injury. One of the proteases that is presumably responsible for this process is matrix metalloproteinase-9 (MMP-9). The levels of MMP-9 are elevated in rodent brain tissue and human blood samples after TBI. However, no studies have described the influence of MMP-9 on the development of PTE. The present study used controlled cortical impact (CCI) as a mouse model of TBI. We examined the detailed kinetics of MMP-9 levels for 1 month after TBI and observed two peaks after injury (30 min and 6 h after injury). We tested the hypothesis that high levels of MMP-9 predispose individuals to the development of PTE, and MMP-9 inhibition would protect against PTE. We used transgenic animals with either MMP-9 knockout or MMP-9 overexpression. MMP-9 overexpression increased the number of mice that exhibited TBI-induced spontaneous seizures, and MMP-9 knockout decreased the appearance of seizures. We also evaluated changes in responsiveness to a single dose of the chemoconvulsant pentylenetetrazol. MMP-9-overexpressing mice exhibited a significantly shorter latency between pentylenetetrazol administration and the first epileptiform spike. MMP-9 knockout mice exhibited the opposite response profile. Finally, we found that the occurrence of PTE was correlated with the size of the lesion after injury. Overall, our data emphasize the contribution of MMP-9 to TBI-induced structural and physiological alterations in brain circuitry that may lead to the development of PTE.

## Introduction

Traumatic brain injury (TBI) is caused by an external mechanical force, such as a blow to the head, concussive forces, acceleration-deceleration forces, or blast injury [1]. One of the major long-lasting consequences of TBI is posttraumatic epilepsy (PTE), which has been estimated to account for 10–20% of symptomatic epilepsies in the general population and 5% of all epilepsy patients [2]. Posttraumatic epilepsy results from molecular and cellular changes that drive the inhibition-excitation balance toward excitation [3]. The molecular and cellular changes that occur after the brain insult also involve the extracellular matrix (ECM), which plays a major role in remodeling neuronal connections after injury. One of the proteases that is implicated in ECM remodeling and consequently synaptic plasticity is matrix metalloproteinase-9 (MMP-9) [4]. MMP-9 is an extracellularly/pericellularly operating protease [5, 6] that regulates numerous cell activities, such as cell differentiation, cell migration, cytokine release, survival, apoptosis, inflammation, and cell-cell contacts [7–9]. In the brain, MMP-9 is expressed and released by neurons and glia, with very low levels in the resting state and markedly greater activity in response to physiological stimulation and various pathological insults [10]. Importantly, MMP-9 levels are increased in the cerebral cortex and hippocampus in mice after TBI [11, 12] and in blood plasma and serum in TBI patients [13, 14]. MMP-9-related synaptic plasticity has been shown to play an important role in the development of epilepsy in both humans and rodents [15, 16]. MMP-9 deficiency inhibits epileptogenesis, and excess MMP-9 facilitates it [17, 18]. Notably, prolonged seizures are related to high serum MMP-9 levels in humans [19]. However, no direct evidence that links MMP-9 to PTE has been reported.

The present study utilized the controlled cortical impact (CCI) model of TBI in mice. After validating the model of CCI-induced structural changes, we investigated the detailed kinetics of enzymatic MMP-9 activity following brain injury in mice. We then evaluated the effects of either MMP-9 knockout or overexpression on neuronal excitability in the cerebral cortex in mice post-trauma. We additionally characterized spontaneous seizure activity in mice 14 weeks following brain trauma. To shed light on the anatomical changes that lead to PTE, we correlated lesion volume with epilepsy morbidity. The results indicated that TBI-induced increases in MMP-9 levels are involved in PTE.

## Materials and Methods

**Fig. 1:**
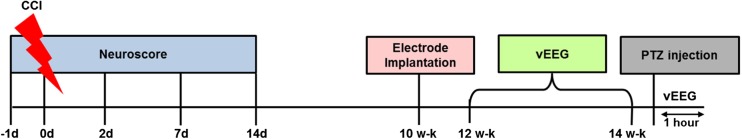
Study design. Somatomotor performance, indicated by neuroscores, was assessed 1 day before TBI and 2, 7, and 14 days after TBI. Skull electrodes were implanted at 10 weeks post-TBI. vEEG monitoring lasted for 2 weeks, starting 14 weeks post-TBI. At 16 weeks post-TBI, the mice were injected with pentylenetetrazol (PTZ) and monitored by vEEG for 1 h

Detailed experimental scheme is presented in Fig. 1.

### Animals

The experiments were performed with adult male C57BL/6J mice (12–14 weeks old; Jackson Laboratory, Bar Harbor, ME, USA). The analysis of the influence of MMP-9 expression levels on lesion volume and the development of epilepsy was performed using mice with modifications of *mmp-9* gene expression. Two transgenic strains were used: homozygous MMP-9 knockout mice on a C57BL/6J background (MMP-9 KO mice) and their wild-type (WT) littermates [20] and mice that overexpressed human pro-MMP-9 under the human PDGF-B promoter on a C57BL/6J background (MMP-9 OE mice) and their WT littermates [21]. Strain colonies (C57BL/6J, MMP-9 KO, and MMP-9 OE) were maintained in the Animal House of the Nencki Institute. Before the experiment, the animals were housed in individual cages under a controlled environment (22 °C ± 1 °C, 50–60% humidity, 12 h/12 h light/dark cycle), with free access to food and water. All of the procedures were performed in accordance with the Animal Protection Act in Poland (directive 2010/63/EU) and were approved by the 1st Local Ethics Committee (permissions no. 383/2012 and 609/2014).

The following numbers of animals comprised the groups in the PTE experiments: MMP-9 KO (*n* = 15), MMP-9 WT (*n* = 10), MMP-9 OE (*n* = 8), MMP-9 WT-OE (*n* = 6). For the studies of the occurrence of seizures, the number of MMP-9 KO animals was doubled because only a small proportion of the mice developed seizures in the first round of the experiment.

### Induction of TBI with CCI

**Fig. 2:**
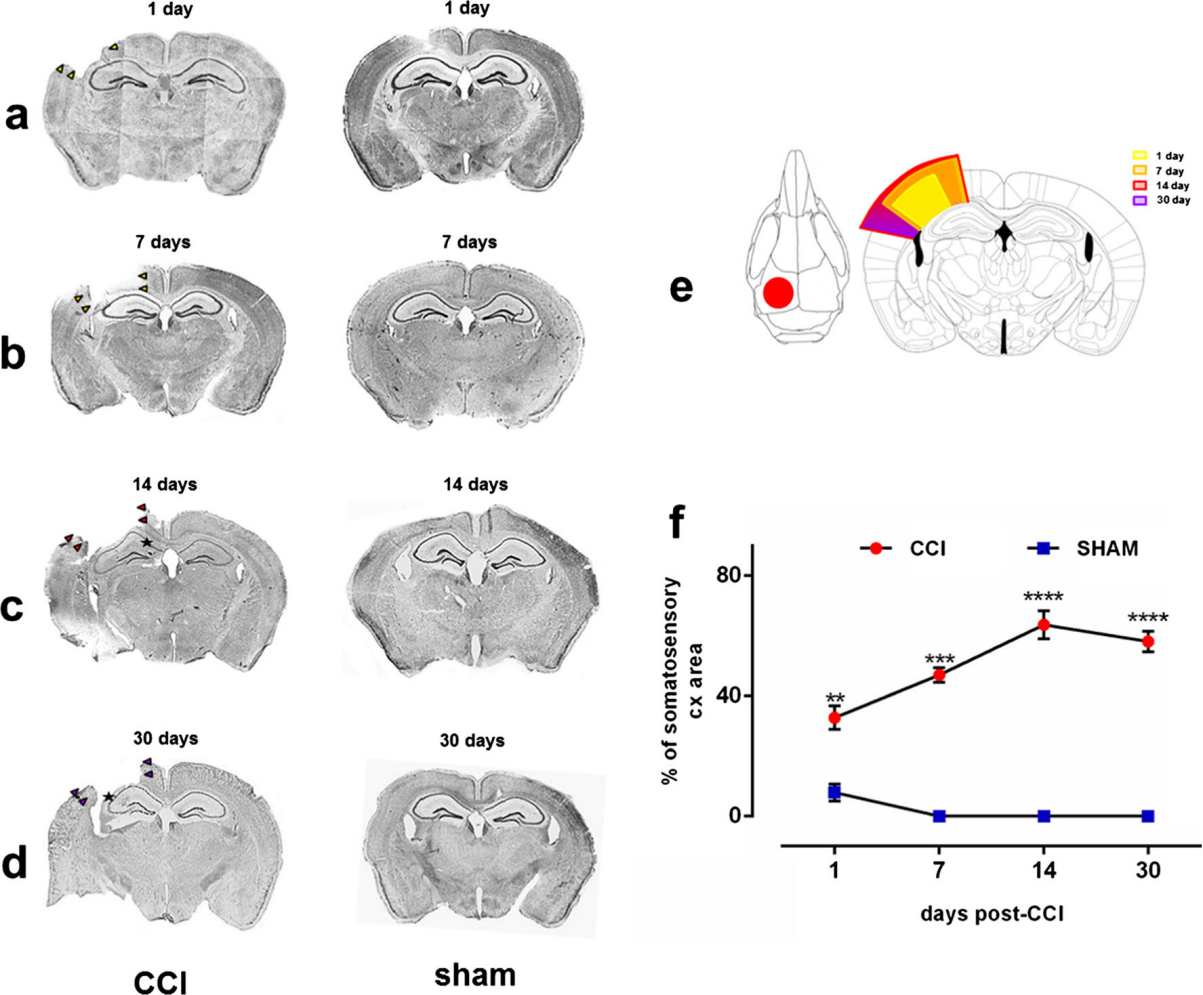
Progressive cerebral cortex degeneration after controlled cortical impact (CCI). **a**–**d** Nissl-stained brain sections from 1 day post-CCI until 30 days post-CCI. CCI, animals after CCI; sham, animals subjected to craniectomy without cortical injury. **e** Craniectomy area, showing a schematic representation of the regions that were analyzed for the progression of cortex damage in CCI-injured mice. Colors indicate days post-injury. **f** Summary of progression of cortex degeneration in CCI-injured mice and sham-operated animals for 30 days post-CCI. The changes were measured as a ratio of lesion volume to the somatosensory cortex (cx) area (%). The data are expressed as mean ± SD. **p < 0.01, ***p < 0.001, ****p < 0.0001 (one-way ANOVA followed by Tukey post hoc test)

The mice were subjected to unilateral cortical contusion using the CCI protocol [22]. The animals were anesthetized with 4% isoflurane (Aerrane, Baxter, UK) in 100% oxygen at a flow rate of 4 L/min and placed in a stereotaxic frame. During surgery, they were maintained with 3% isoflurane and 100% oxygen at a flow rate of 0.6 L/min (Combi Vet Anesthesia System, Rothacher, Switzerland). For deeper sedation, the mice were injected with butorphanol (10 μg/30 g body weight). The skull was exposed by a midline scalp incision, and craniectomy was performed using a 5 mm ∅ trephine (Fine Science Tools FST, Heidelberg, Germany) over the left parietotemporal cortex between lambda and bregma (Fig. 2e, 5a1). The bone piece was carefully removed without disruption of the underlying dura. Traumatic brain injury was induced with a Leica Impact One device (Leica Biosystems, Kawaska, Poland) that was equipped with an electrically driven metallic piston that was controlled by a linear velocity displacement transducer. After craniectomy, the adjustable equipment for CCI was mounted on the left stereotaxic arm at a 20° angle from vertical. CCI was performed per protocol using the following parameters: 3 mm ∅ (flat tip), 0.5 mm depth from dura, 5 m/s velocity, and 100 ms dwell time. After the injury, bleeding was controlled. A piece of sterile plastic was placed over the craniectomy, and the incision was sutured with nylon stitches (Sigmed, Cisek, Poland). The animals were then returned to heated home cages for postsurgical recovery. Sham-injured animals (*n* = 3 for each experiment per time point) underwent identical anesthesia and craniectomy procedures but were not subjected to CCI.

### Nissl Staining

The mice were anesthetized and perfused with 0.37% sulfide solution (5 ml/min, 4 °C) for 5 min, followed by 4% paraformaldehyde in 0.1 M sodium phosphate buffer (pH 7.4, 5 ml/min, 4 °C) for 10 min. The brains were removed from the skull, postfixed in buffered 4% paraformaldehyde for 4 h at 4 °C, and then cryoprotected in a solution that contained 30% glycerol in 0.02 M potassium phosphate-buffered saline (PBS) for 48 h. The brains were then frozen on dry ice and stored at − 80 °C. Frozen brains were sectioned in the coronal plane (40 μm) with a sliding cryostat (Leica Biosystems, Kawaska, Piaseczno, Poland). The sections were mounted on gelatin-covered microscope slides, dried, and stained with Cresyl Violet. Pictures of Nissl-stained sections were taken using a Nikon Eclipse Ni light microscope that was equipped with a PlanApo 2× objective.

### Gel Zymography

Gel zymography of tissue that was isolated from the ipsi- and contralateral cortex and hippocampus in CCI and sham C57BL/6J mice was performed according to Szklarczyk et al. [23]. After 10 min, 30 min, 60 min, 2 h, 6 h, 1 day, 3 days, 7 days, 14 days, and 30 days, the brains were rapidly removed, and tissue was dissected on a cold plate and frozen on dry ice. Each time-point group consisted of three CCI animals and three sham-operated animals. The samples were stored at − 80 °C until analysis. After tissue frostbite, the samples were homogenized in a buffer that contained 10 mM CaCl_2_, 0.25% Triton X-100, and protease inhibitor cocktail (c*O*mplete mini EDTA-free; Roche, Basel, Switzerland) and centrifuged at 6000×*g* for 30 min. The entire supernatant that contained soluble proteins was quantitatively recovered. The pellet (Triton X-100-insoluble) was resuspended in a buffer that contained 50 mM Tris (pH 7.4) and 0.1 M CaCl_2_ in water, heated for 15 min at 60 °C, and then centrifuged at 10,000×*g* for 30 min at 4 °C. This treatment results in the release of ECM-bound MMPs into the solution. The final pellet was free from MMP activity, as evaluated by gel zymography, thus confirming completeness of the extraction. The final supernatant was considered a Triton X-100-insoluble fraction. After centrifugation, the entire supernatant was quantitatively recovered. Sample protein concentrations were measured using the BCA protein assay (Pierce, Rockford, IL, USA). After quantification, samples that were lysed in buffer without 2-mercaptoethanol were subjected to sodium dodecyl sulfate-polyacrylamide gel electrophoresis with 8% Tris-glycine acrylamide gels that contained 0.5% gelatin (Sigma-Aldrich, St. Louis, MO, USA) under nondenaturating and nonreducing conditions. The gels were washed twice for 30 min each in 2.5% Triton X-100 and incubated in zymography buffer (50 mM Tris [pH 7.5], 10 mM CaCl_2_, 1 M ZnCl_2_, 1% Triton X-100, and 0.01% sodium azide) for 5–7 days and stained with 0.5% Coomassie blue G-250 (Sigma-Aldrich, St. Louis, MO, USA). The optical density (intensity) of white bands on a blue background corresponded to MMP-9 levels and was quantified with the GeneTool program. For MMP-9 level comparisons between tissue and blood, we collected venous serum samples from CCI and sham animals. Blood was collected on 3.2% sodium citrate and centrifuged at 13,000×*g* for 30 min at 4 °C. Serum was then collected and frozen at − 80 °C for further analysis.

### Neuroscore Test Following TBI

After CCI, the mice were subjected to behavioral testing to assess their level of motor and cognitive functions. The assessment of motor function was performed using the neuroscore test as previously described by Scherbel et al. [24]. The test began 1 day before TBI to obtain a baseline score and was repeated on days 2, 7, and 14 following TBI. The 20-point composite neuroscore was derived from the sum score of three tests: forelimb and hindlimb flexion tests (0–4 score for individual forelimbs/hindlimbs, maximum score = 8) and angle board test (0–4 score, maximum score = 4). In the forelimb flexion test, forelimb coordination and grip strength were evaluated when the mouse was suspended from its tail over a metal cage top and then lowered to allow it to grasp the cage bars with both forelimbs. One point was deducted from the forelimb score if the mouse exhibited a reduction of grip strength, crossed its forelimbs, or presented excessive hyperactivity and limb spasms. In the hindlimb flexion test, abnormal limb extension or toe splaying each resulted in a 1-point deduction, whereas three points were deducted if the mouse curled its hindlimb up to its body. The mice were then tested in the angle board test to assess their ability to stand on an inclined plane that was covered with a vertically grooved rubber mat. On the first test day (i.e., 1 day before TBI), the board was inclined at a 40° angle. The mouse was first placed on the board with its head upward, then to the left, then to the right, and finally downward. The mouse had to stay on the board for 5 s without holding on by its tail. The angle of the board was increased in 2.5° increments until the mouse could no longer stand on the board. After TBI, the tests were started at a 10° inclination below the animals’ baseline value, and scores (0–4) were assigned for each direction. A 2.5° decrease from the baseline angle resulted in a 1-point reduction of the angle board score. The neuroscore was used to calculate two additional parameters. ΔImpairment (Δi) refers to the difference in neuroscore between the baseline and day 2 post-TBI. ΔRecovery (Δr) was calculated by subtracting the neuroscore on day 2 post-TBI from the neuroscore on day 14 post-TBI.

### Intracranial Electrode vEEG Monitoring of Epileptiform Activity

Four stainless-steel screw electrodes (1.6 mm ∅, Bilaney Consultants GmbH, Dusseldorf, Germany) were implanted 10 weeks post-TBI. One recording electrode was placed ipsilaterally, rostromedial to the craniectomy. Another recording electrode was placed contralaterally to the region that corresponded to the center of the craniectomy. A reference electrode was positioned above the contralateral frontal cortex. A ground electrode was placed in the occipital bone over the cerebellum (Fig. 5a1). Two weeks of continuous (24 h/day, 7 days/week) video-EEG (vEEG) monitoring began 12 weeks post-TBI [25]. The mice were placed in Plexiglas cages (one mouse per cage) and connected to the recording system with commutators (SL6C, Plastics One, Roanoke, VA, USA). vEEG was performed using the Twin EEG recording system that was connected to a Comet EEG PLUS with AS40-PLUS 57-channel amplifier (Natus Medical, Pleasanton, CA, USA) and filtered (high-pass filter cut-off 0.3 Hz, low-pass filter cut-off 100 Hz). The animals’ behavior was recorded using an I-PRO WV-SC385 digital camera (Panasonic, Osaka, Japan). As outcome measures, we assessed the occurrence, frequency, and duration of spontaneous seizures. An electroencephalographic seizure was defined as a high-amplitude (> 2 times baseline) rhythmic discharge that clearly represented an abnormal EEG pattern that lasted > 5 s. The frequency of seizures for each mouse was calculated as the number of seizures per completed EEG recording day or per week. The modified 0–5 point Racine scale was used: 0 (electrographic seizure without any detectable motor manifestation), 1 (mouth and face clonus, head nodding), 2 (clonic jerks of one forelimb), 3 (bilateral forelimb clonus), 4 (forelimb clonus and rearing), and 5 (forelimb clonus with rearing and falling) [26]. The mice were monitored for the appearance of spontaneous seizures during the monitoring period. Mice with modified levels of MMP-9 were used and randomly assigned to the following groups: MMP-9 KO mice (*n* = 15), WT littermates of MMP-9 KO mice (*n* = 10), MMP-9 OE mice (*n* = 8), and WT littermates of MMP-9 OE mice (*n* = 6).

### Pentylenetetrazol Threshold Test

After 2 weeks of vEEG recording (14 weeks after CCI), the mice were examined for pentylenetetrazol (PTZ)-induced seizure susceptibility. The animals were randomly assigned to groups. The first part of the experiment included C57BL/6J mice, 14 weeks after CCI (*n* = 13), and sham animals (*n* = 5). The second part of the experiment included mice with modified levels of MMP-9: MMP-9 KO mice (*n* = 15), WT littermates of MMP-9 KO mice (*n* = 10), MMP-9 OE mice (*n* = 8), and WT littermates of MMP-9 OE mice (*n* = 6). To test for seizure susceptibility at 14 weeks post-CCI (in the C57BL/6J and sham groups) or on the last day of vEEG recording (MMP-9 KO, MMP-9 WT, MMP-9 OE, and MMP-9 WT-OE groups), the animals were intraperitoneally injected with a subconvulsant dose of PTZ (30 mg/kg; Sigma-Aldrich, St. Louis, MO, USA) [27]. Immediately following the single injection of PTZ, the mice were connected to the monitoring system and observed for 60 min. The latency (in seconds) between the injection and the first epileptiform discharge (Fig. 7a-b) was recorded.

### Traumatic Lesion Volume Quantification

Fourteen weeks post-CCI, four groups of mice (MMP-9 KO [*n* = 5], MMP-9 WT [*n* = 5], MMP-9 OE [*n* = 5], and MMP-9 WT-OE [*n* = 5]) were transcardially perfused with chilled (4 °C) PBS, followed by 4% paraformaldehyde in 0.1 M PBS. After staining the coronal sections with Cresyl violet, photographs were taken with a Nikon Eclipse Ni light microscope with a PlanApo 2 × 0.1 objective using ImagePro Plus 7.0 software. The histological lesion area was quantified with ImageJ software and is presented as a ratio between the lesion area and somatosensory cortex. To confirm the functionality of the model, gel zymography was performed in unstimulated (naive) and stimulated (24 h after brain injury) animals to evaluate MMP-9 levels in mice with modified genotypes.

### Statistical Analyses

The statistical analysis was performed using GraphPad Prism 6.0 software. Dynamic cortex degeneration upon CCI and differences in lesion area were compared using one-way analysis of variance (ANOVA) followed by Tukey’s multiple-comparison test. The level of active MMP-9 was analyzed using two-way ANOVA followed by the Sidak post hoc test. Neuroscores were analyzed using the nonparametric Mann-Whitney *U* test. Differences between injured animals and sham animals in PTZ-induced seizure thresholds were analyzed using the nonparametric Mann-Whitney *U* test or one-way ANOVA followed by the Sidak post hoc test. Differences in epileptiform activity 3.5 months post-CCI between genotypes were analyzed using one-way ANOVA followed by the Sidak post hoc test. Values of *p* < 0.05 were considered statistically significant.

## Results

### Time-Dependent Somatosensory Cortex Degeneration and Long-Term Motor Function After CCI-Induced TBI

As a model of TBI, we used CCI in mice [28]. To characterize CCI-induced brain injury, brains were collected at different time-points after the insult (1, 7, 14, and 30 days). Sham-operated control animals were subjected to craniectomy only. Massive cerebral cortex degeneration was observed in the injured area beginning on day 1 post-CCI (32% cortical loss). The level of degeneration reached a peak after 14 days (64% cortical loss) and stabilized over next 2 weeks (58% cortex loss) compared with the contralateral site (Fig. 2c–f). The sham groups exhibited only minor cortical changes compared with CCI animals (1 day, *p* = 0.018; 7 days, *p* = 0.0027; 14 days, *p* < 0.0001; 30 days, *p* < 0.0001; Fig. 2a, d, f).

### MMP-9 Is Upregulated After TBI

**Fig. 3:**
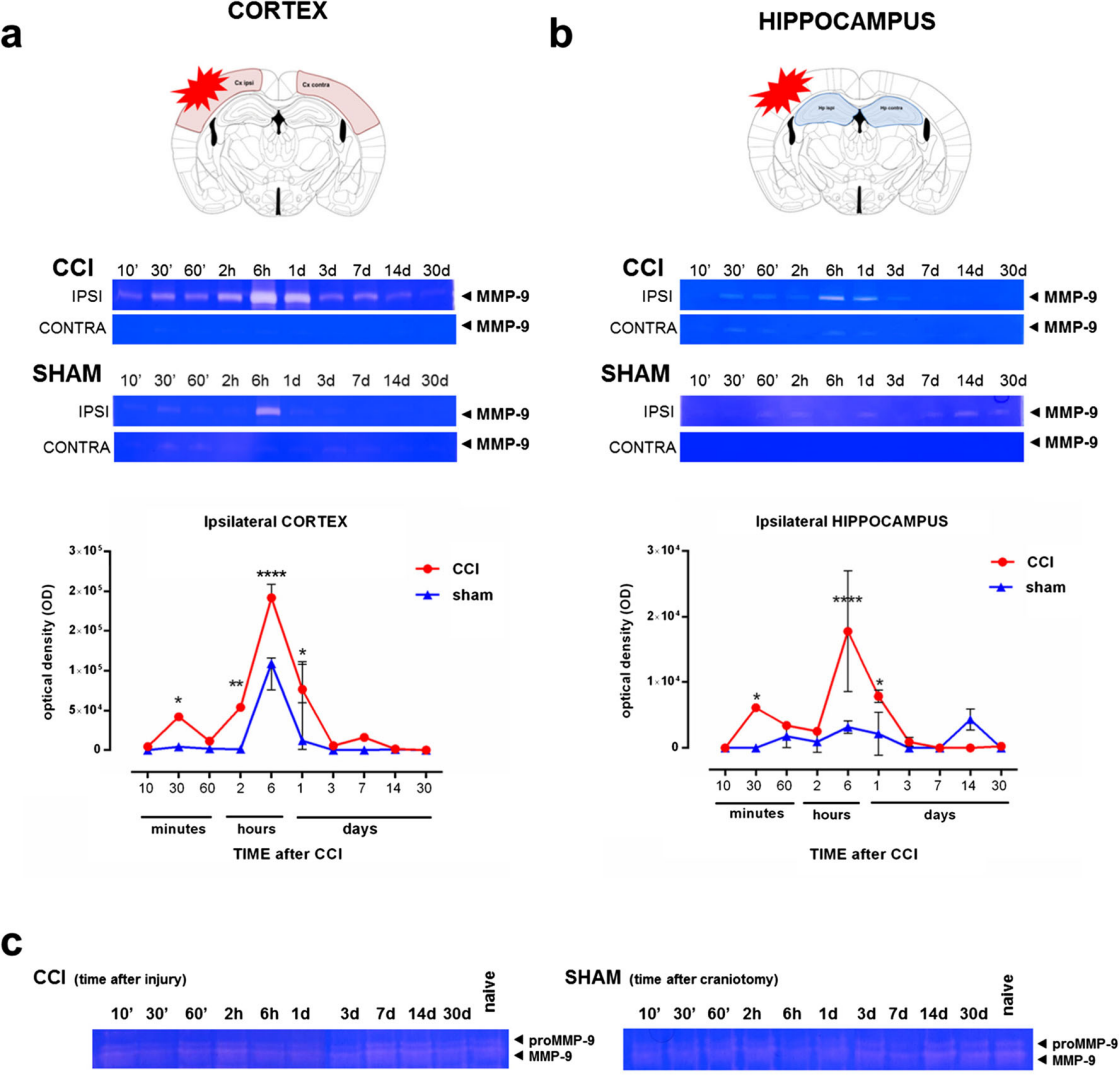
Time-dependent MMP-9 levels in the cerebral cortex and hippocampus after controlled cortical impact (CCI). **a**, **b** Gel zymography from ipsi- and contralateral cortex (**a**) and hippocampus (**b**) performed 10 min, 30 min, 60 min, 2 h, 6 h, 1 day, 3 days, 7 days, 14 days, and 30 days post-CCI. CCI, mice after CCI; sham, mice after craniectomy (without brain damage). **c** Gel zymography of venous blood plasma that was collected from animals after CCI and sham animals at different time-points. Representative zymograms are shown. For zymograms, each time-point group consisted of three CCI animals and three sham animals. MMP-9 levels were measured for each sample separately. The data are expressed as mean ± SD. *p < 0.05, **p < 0.01, ****p < 0.0001 (two-way ANOVA followed by Sidak post hoc test)

To characterize the temporal profile of MMP-9 levels after TBI, brain tissue samples were collected from CCI- and sham-operated animals at 10 min, 30 min, 60 min, 2 h, 6 h, 1 day, 3 days, 7 days, 14 days, and 30 days. The somatosensory cortex and hippocampus were collected from the injured hemisphere (ipsilateral) and contralateral hemisphere separately. Venous blood was collected to measure plasma MMP-9 levels. Gel zymography was performed. Traumatic brain injury that was induced by CCI significantly increased MMP-9 levels in the ipsilateral somatosensory cortex 30 min after CCI (*p* < 0.05; Fig. 3a). MMP-9 levels then decreased slightly 2 h after CCI and rose again 6 h after CCI, with a two-fold increase compared with sham-operated animals (*p* < 0.0001; Fig. 3a). Interestingly, MMP-9 levels also increased in sham-operated animals 6 h post-CCI and then began to decrease. However, after 1 day, MMP-9 levels were still elevated (Fig. 3a). Sustained MMP-9 levels were observed over the next 2 weeks (i.e., between days 3 and 14 post-TBI; Sidak post hoc test; time factor: *F*_9,36_ = 49.66, *p* < 0.0001; injury factor: *F*_1,4_ = 96.31, *p* = 0.0006; Fig 3a).

A similar but weaker effect was observed in the hippocampus, where MMP-9 levels were elevated after 30 min (*p* < 0.05; Fig. 3b). Peak MMP-9 levels were observed after 6 h (five-fold increase compared with sham-operated group; *p* < 0.0001; Fig. 3b), and they decreased during the next days. Interestingly, we found significant heterogeneity between animals in the CCI group at the 6 h time-point. In the contralateral hemisphere, MMP-9 was nearly undetectable (Fig. 3a, b). In sham-operated animals, craniectomy alone did not increase MMP-9 levels in the hippocampus (Sidak post hoc test; time factor: *F*_9,36_ = 11.72, *p* < 0.0001; injury factor: *F*_1,4_ = 17.78, *p* = 0.0135).

No significant changes in plasma MMP-9 levels upon brain injury (Fig. 3c) were found between the CCI, sham, and naive groups (without any manipulations).

### MMP-9 Levels in Genetically Modified Animals

**Fig. 4:**
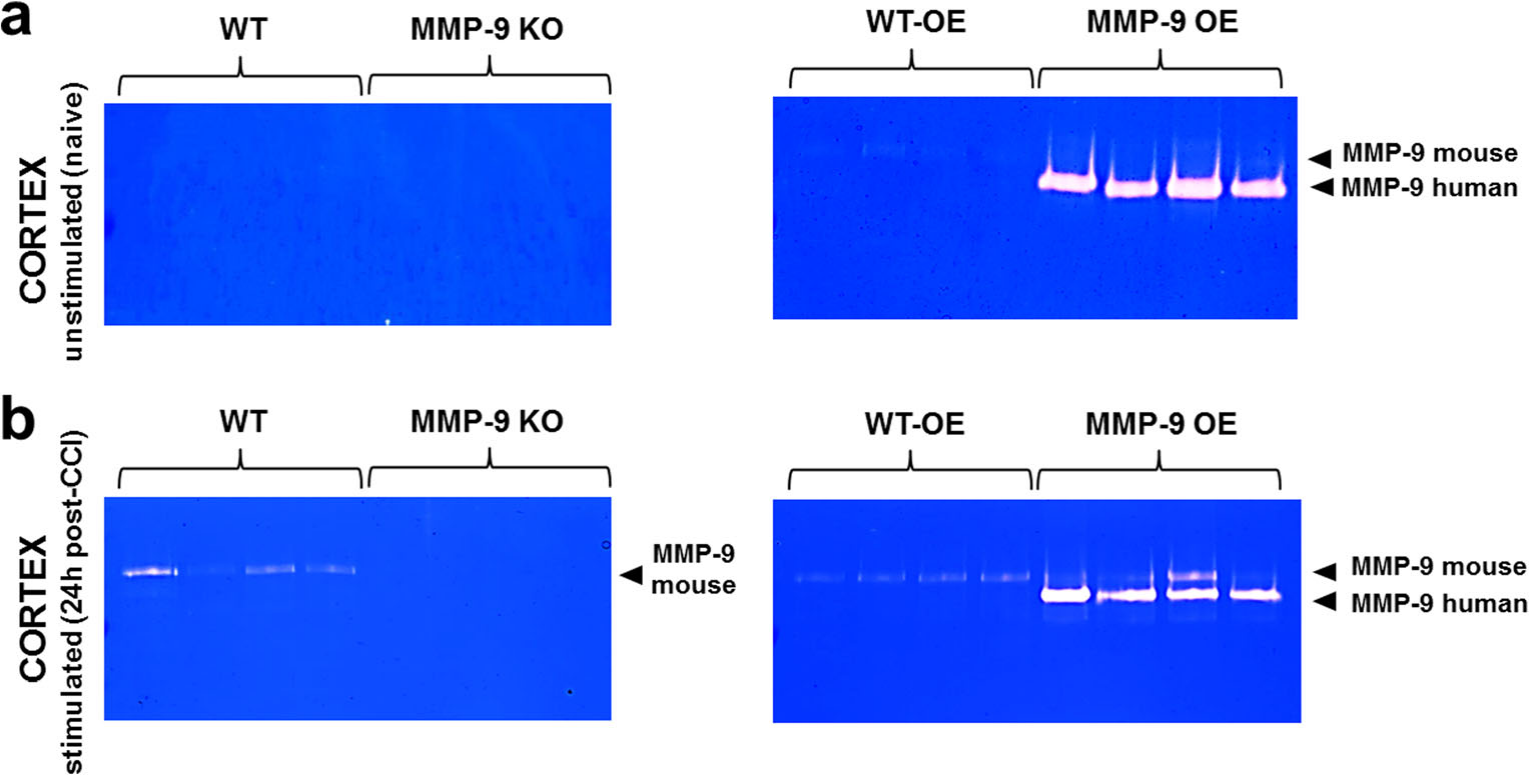
Gel zymography of MMP-9 levels in the cortex induced by CCI. **a** Zymograms from the cortex in unstimulated (naive) animals with modification of *mmp-9* gene expression levels. WT and MMP-9 KO (left). WT-OE and MMP-9 OE (right). **b** Gel zymography of MMP-9 levels in the cortex in stimulated mice (24 h post-CCI) with modification of *mmp-9* gene expression levels. WT and MMP-9 KO (left). WT-OE and MMP-9 OE (right)

To investigate the functional role of MMP-9 in the development of PTE after TBI, we used MMP-9 KO and MMP-9 OE mice. Each genotype had its own WT littermate control group. We assessed MMP-9 levels by gel zymography. We also analyzed MMP-9 levels before and after the brain insult (24 h post-CCI; Fig. 4a; *n* = 4/group). Basal activity in the ipsilateral cortex in naive animals was detected only in MMP-9 OE animals. No gelatinolytic MMP-9 activity was observed in the MMP-9 KO, MMP-9 WT, or MMP-9 WT-OE group (Fig. 4a). The CCI increased endogenous MMP-9 levels in MMP-9 WT, MMP-9 OE, and MMP-9 WT-OE groups. No changes in MMP-9 levels were observed in the MMP-9 KO group (Fig. 4b). After stimulation in the MMP-9 OE group, we observed MMP-9 of both mouse origin (92 kDa) and MMP-9 that derives from the human pro-MMP-9 genomic insert (lower band in Fig. 4b).

### No Genotype-Dependent Differences in Motor Activity After Brain Injury

**Fig. 5:**
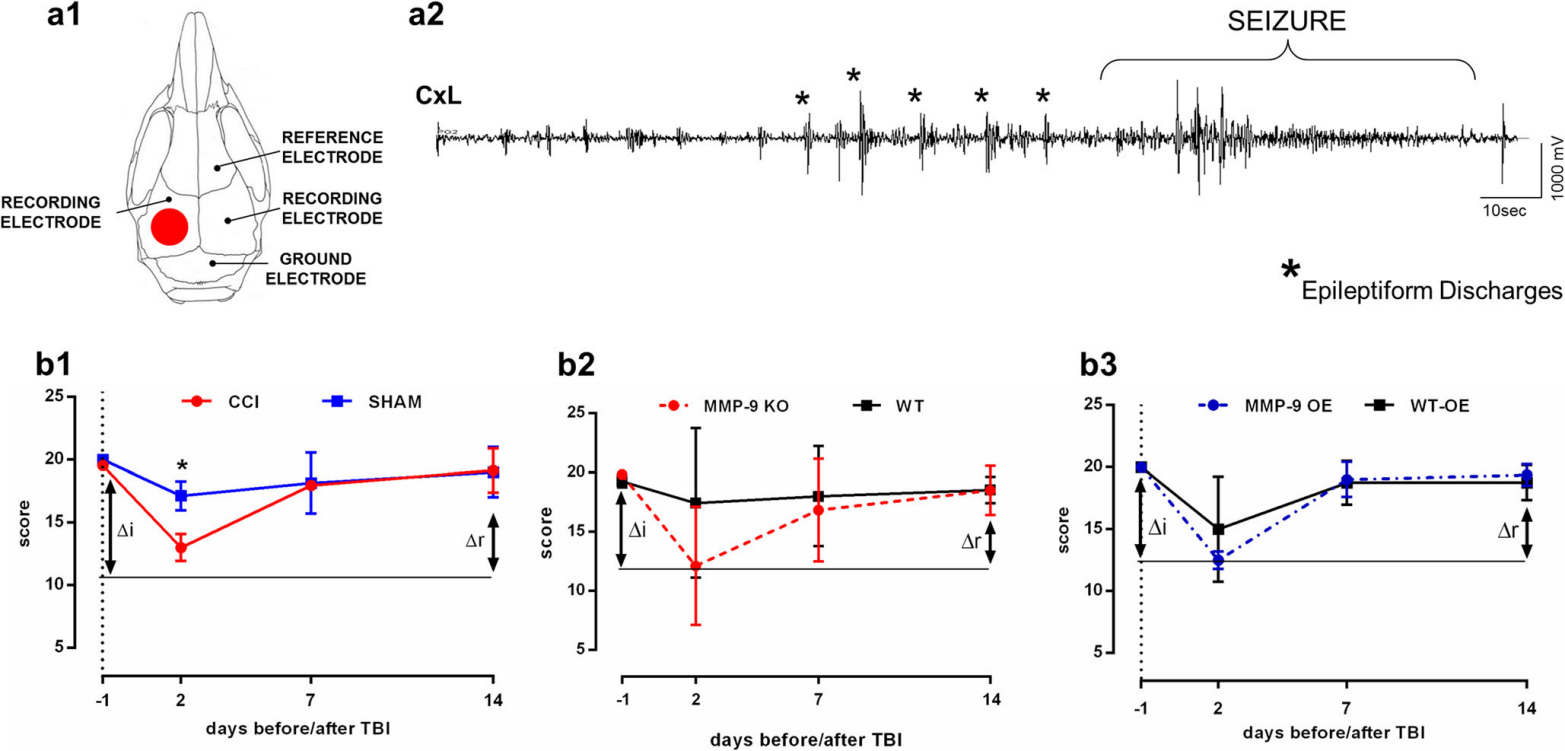
**a1** Location of craniectomy (red circle) and placement of skull electrodes. **a2** Example of spontaneous seizure in the ipsilateral perilesional cortex (CxL) that lasted > 50 s (behavioral score = 5) preceded by epileptiform discharges (*) that lasted < 2 s. **b1**–**3** Assessment of somatomotor performance, indicated by neuroscores, in mice with modification of *mmp-9* gene expression. ∆i, impairment (difference between baseline on day − 1 and day 2 post-TBI); ∆r, recovery (difference between scores on days 2 and 14 post-TBI). The data are expressed as mean ± SD. Changes between groups were assessed each day separately. *p < 0.05 (nonparametric Mann-Whitney test).

To clarify the experimental results, we employed a study design that was based on Miszczuk et al. [29] (Fig. 1). Following TBI, the mice were subjected to behavioral testing to assess their level of motor function. Animals that were subjected to CCI were tested for motor recovery, based on neuroscores, 1 day before and 2, 7, and 14 days post-injury [18]. The animals were put on an angled platform, and motor performance was tested, based on the angle of inclination. The mice received scores for stability in four directions (head up, down, left, and right). All of the scores were compared with basal scores that were recorded 1 day before the injury. Motor recovery in CCI mice was impaired compared with the sham groups 2 days after CCI (Fig. 5b1; *p* = 0.0133), and then all animals fully recovered 7 days after CCI. No significant differences in ∆i (difference between baseline on day − 1 and day 2 post-TBI; *p* = 0.2) or ∆r (recovery; difference between the scores on days 2 and 14 post-TBI; *p* = 0.2) were observed between the CCI and sham groups.

To verify the effect of genotype, analogical analysis was performed in animals with *mmp-9* gene modification (Fig. 5b2, b3). We did not observe any significant differences between MMP-9 KO mice and MMP-9 WT mice after CCI (day 2 post-CCI; *p* = 0.2487), with no significant differences in ∆i (*p* = 0.1907) or ∆r (*p* = 0.0985). Similarly, no differences were observed between MMP-9 OE mice and MMP-9 WT-OE mice after CCI (day 2 post-CCI; *p* = 0.333), with no significant differences in ∆i (*p* = 0.333) or ∆r (*p* = 0.2667). We found motor impairment between MMP-9 KO and MMP-9 WT mice and between MMP-9 OE and MMP-9 WT-OE mice during the first 24 h after injury. Motor activity returned to basal levels within the next 6 days.

### MMP-9 Contributes to the Development of Epilepsy

**Fig. 6:**
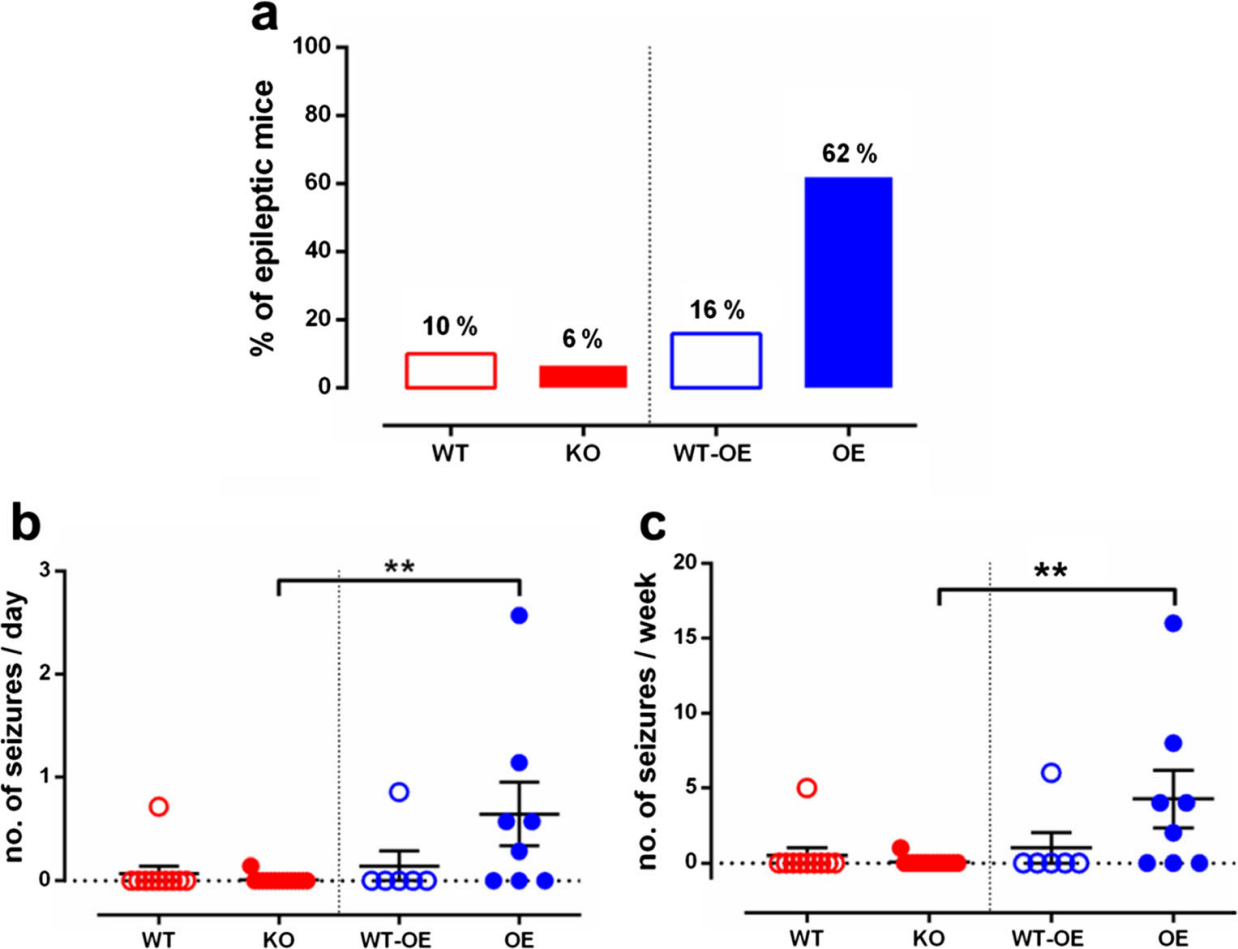
Epileptiform activity in MMP-9 KO mice and MMP-9 OE mice during the 3.5 months of follow-up after controlled cortical impact-induced traumatic brain injury. Data from the vEEG recordings were collected between weeks 12 and 14 after injury. **a** Percentage of epileptic animals that developed a minimum of one spontaneous seizure per 2 weeks of recordings. **b** Number of seizures per day. **c** Number of seizures per week. The data are expressed as mean ± SD. *p < 0.05, **p < 0.01 (one-way ANOVA followed by Sidak post hoc test)

Ten weeks after CCI, the mice were implanted with electrodes (Fig. 5a1). After 1 week of recovery, vEEG monitoring was performed for 2 weeks. The number of epileptic animals and number of seizures per day and per week were recorded. MMP-9 deficiency protected animals against the development of spontaneous seizures. Only 6% (1/15) of MMP-9 KO mice exhibited spontaneous seizures compared with MMP-9 WT mice (10%; 1/10), whereas 62% (5/8) of MMP-9 OE mice exhibited the epileptic phenotype (Fig. 6a). Among the animals that developed spontaneous seizures, the number of seizures per week of recording differed between MMP-9 KO and MMP-9 OE mice. MMP-9 KO mice had fewer seizures per week (*p* = 0.0017) compared with MMP-9 OE mice (Sidak post hoc test; genotype effect: *F*_3,35_ = 4.351, *p* = 0.0105) (Fig. 6b). Similarly, MMP-9 KO mice exhibited fewer seizures per day (*p* = 0.0017) than MMP-9 OE mice (Sidak post hoc test; genotype effect: *F*_3,35_ = 4.124, *p* = 0.0132) (Fig. 6a).

### MMP-9-Dependent Seizure Susceptibility Evoked by Single Subconvulsant Dose of Pentylenetetrazol

**Fig. 7:**
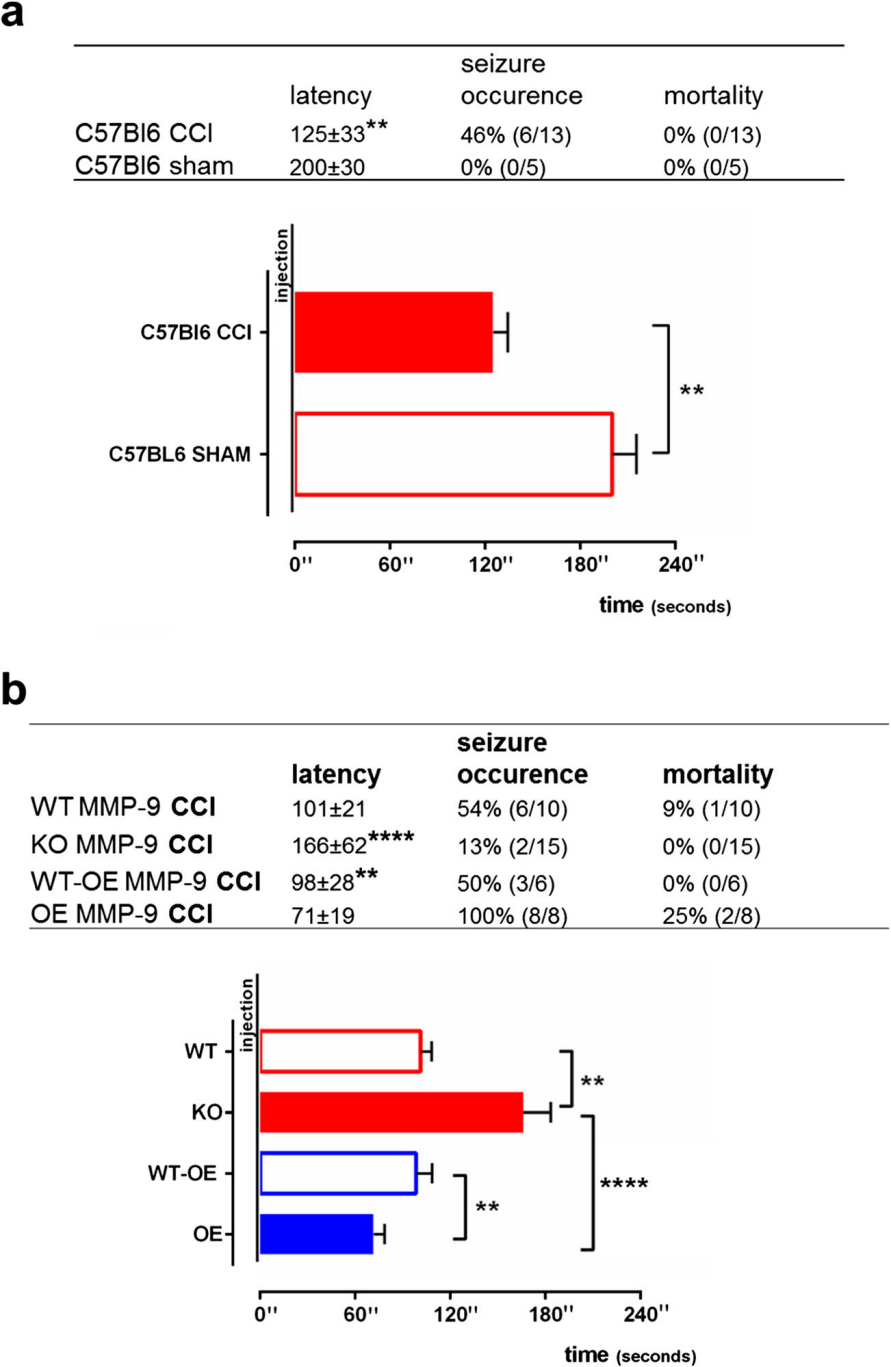
Post-CCI neuronal excitability in the pentylenetetrazol threshold test. A subconvulsant dose of PTZ (30 mg/kg) was injected on the last day of vEEG recording (14 weeks post-CCI). **a**, **b** Latency between the PTZ injection and first epileptiform discharge measured in C57BL/6J and sham mice (**a**) and mice with modification of MMP-9 levels (**b**). The table presents the latency (in seconds), seizure occurrence (% of animals), and mortality (%). The data are expressed as mean ± SD. **p < 0.01, ****p < 0.0001 (nonparametric Mann-Whitney test for C57BL/6J animals, one-way ANOVA followed by Sidak post hoc test for animals with modification of *mmp-9* gene expression)

On the last day of vEEG, the mice received an intraperitoneal injection of a subconvulsant dose of PTZ (30 mg/kg). We first evaluated the influence of TBI on PTZ-induced seizure susceptibility. We used C57BL/6J control animals after CCI and sham-operated littermates and injected PTZ 12 weeks post-CCI/sham surgery. We observed a significantly shorter latency between the PTZ injection and the first epileptiform discharge in CCI animals compared with sham-operated animals (*p* = 0.0017; Fig. 7a) and a higher occurrence of seizures (46%).

To evaluate the effect of MMP-9 on PTZ-induced seizure susceptibility, we used MMP-9 KO and MMP-9 OE mice. The latency between the PTZ injection and first epileptiform discharge was significantly shorter in MMP-9 KO mice compared with MMP-9 WT mice (*p* = 0.0022; Fig. 7b) and MMP-9 OE mice (*p* < 0.0001; Fig. 7b). The occurrence of seizures in MMP-9 KO mice was significantly less frequent (15%) compared with MMP-9 OE mice (100%). Pentylenetetrazol-induced seizures were observed in 54% of MMP-9 WT mice and 50% of MMP-9 WT-OE mice. Mice with higher levels of MMP-9 had a higher mortality rate after the PTZ injection.

### Post-CCI Lesion Volume Is Dependent on MMP-9 Levels

**Fig. 8:**
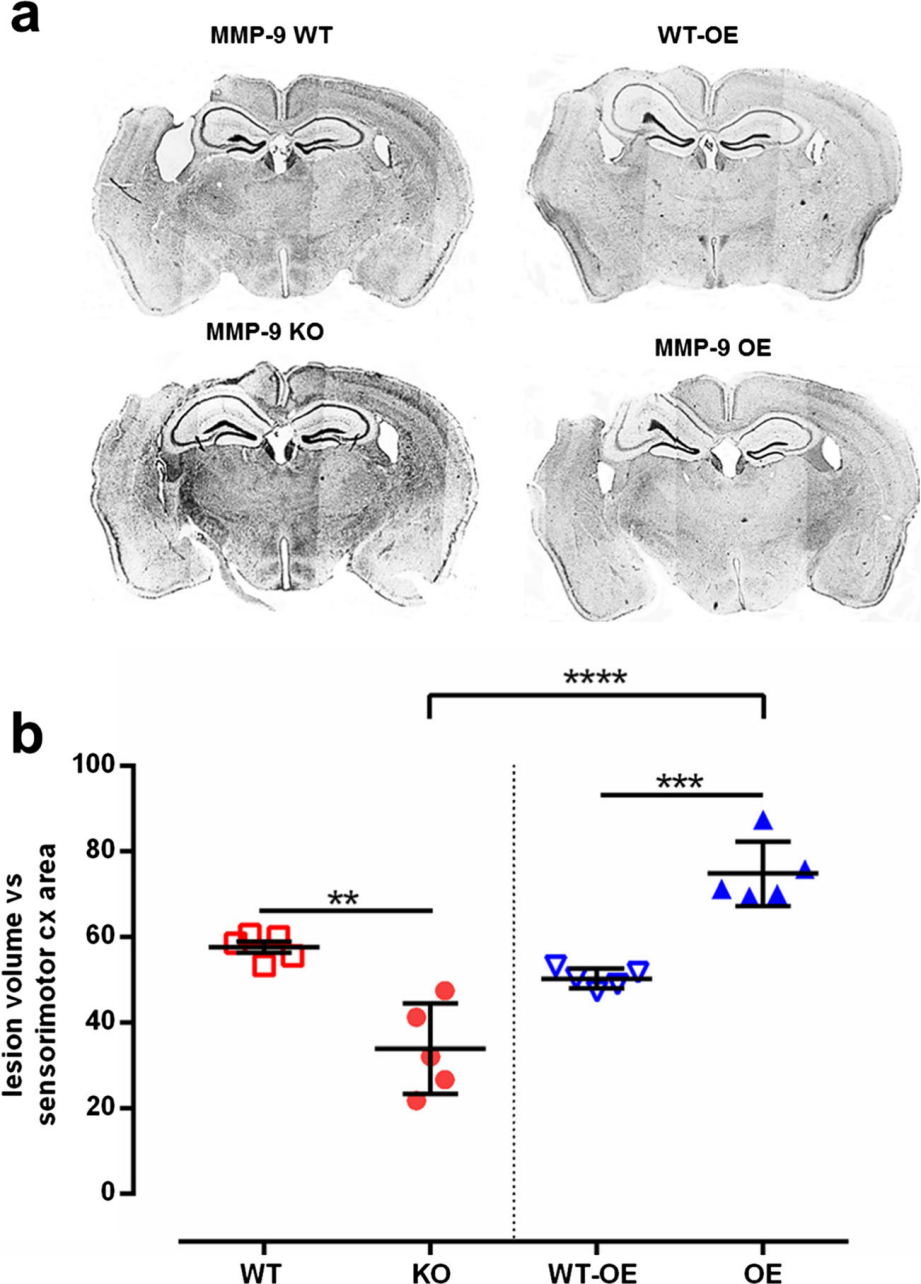
Influence of genetic modification of MMP-9 levels on post-CCI cerebral cortex lesion volume. **a** Example sections from mice at 14 weeks post-CCI, stained with Cresyl violet. **b** Lesion volume in mice with modification of *mmp-9* gene expression. The brain sections were analyzed using ImageJ software. The lesion area is presented relative to the somatosensory cortex area. The data are expressed as mean ± SD. **p < 0.01, ***p < 0.001, ****p < 0.0001 (one-way ANOVA followed by Tukey post hoc test)

Finally, we correlated MMP-9 levels with lesion severity. Fourteen weeks after CCI, brain tissue was collected, and sections were stained with Nissl staining to assess the size of the lesion after injury. MMP-9 KO mice had a smaller lesion volume within the injury area compared with MMP-9 WT mice (*p* < 0.01; Fig. 8a, b); MMP-9 OE mice had a significantly greater lesion volume than MMP-9 WT-OE mice (*p* < 0.001; Fig. 8a, b). The lesion volume in MMP-9 KO mice was significantly smaller compared with MMP-9 OE mice (genotype effect: *F*_3,16_ = 31.88, *p* < 0.0001).

## Discussion

In the present study, TBI significantly increased MMP-9 levels in the perilesional cortex and ipsilateral hippocampus 30 min, 6 h, and 24 h post-injury. Elevations of MMP-9 levels augmented both the susceptibility to PTZ-induced seizures and occurrence of spontaneous seizures. Deficiency of the active form of the MMP-9 in MMP-9 KO mice reduced epileptogenesis. We also found that MMP-9 KO mice had smaller post-TBI cortical lesion volumes, whereas MMP-9 OE mice had greater lesion volumes in the cerebral cortex.

Previous studies of time-dependent increases in MMP-9 levels post-injury focused on the events that occurred 1–7 days after the induction of brain damage [11, 12, 30]. However, little is known about MMP-9 levels during the acute phase after TBI (i.e., within the first 24 h) and during the chronic phase up to several weeks post-TBI when epileptic seizures might start to occur. Therefore, the present study extended this time-course by measuring MMP-9 levels both during the acute phase post-CCI throughout the first 24 h and during the chronic phase up to 30 days post-injury. Brain injury increased active MMP-9 levels in the cerebral cortex and hippocampus within the first hour post-CCI. This increase was followed by a decrease in MMP-9 levels after 1–2 h and then a marked increase in MMP-9 levels between 6 and 24 h after the cortical insult. This time-course of elevations of MMP-9 levels was observed most prominently in the cerebral cortex that surrounded the injury and to a lesser extent in the ipsilateral hippocampus. The dynamic changes in MMP-9 can be explained by complex and multi-stage mechanisms of MMP-9 expression and activation. Neuronal MMP-9 can be produced within minutes after excitatory simulation through local dendritic/synaptic translation from the preexisting pool of mRNA [31]. Within 2 h following stimulation, transcription-dependent MMP-9 accumulation can occur [32]. Under conditions of sustained stimulation, as previously demonstrated following kainate treatment, various molecular mechanisms of MMP-9 production and activation overlap, resulting in a massive and prolonged increase in the levels of the enzyme and its activity [23]. Similar phenomena of sustained MMP-9 expression, translation, release, and activation may occur around the injury site after TBI (i.e., within the surrounding cerebral cortex and hippocampus), with the involvement of neurons, glia, and invading leukocytes as cellular sources of MMP-9.

Interestingly, we did not observe any changes in gelatinolytic MMP-9 levels in blood serum that was collected from injured, sham, and naive animals. This is in contrast with human studies, in which high MMP-9 and MMP-2 levels were detected acutely post-injury in blood plasma as well as in brain extracellular and cerebrospinal fluids in adult patients with moderate to severe TBI [33]. In humans, high MMP-9 levels were associated with poorer outcomes, including a longer stay in the intensive care unit and a greater risk of mortality [33]. Severe TBI in humans leads to long-term cognitive and motor dysfunction [34]. Severe injury may cause MMP-9 release from other cells, and such higher levels of MMP-9 may then be detectable in blood in patients. In the present study, neuroscores were used to assess motor activity in mice after CCI, with the deficits being relatively minor, and the animals’ performance in the test quickly returned to basal levels. Therefore, our model appears to produce less severe TBI than the one reported in the aforementioned human study, and thus, it produces less enhanced MMP-9 levels.

The involvement of MMP-9 in epileptogenesis has been previously reported [18, 35–37]. However, to date, the role of MMP-9 in PTE that is evoked by TBI has not been investigated. Therefore, we used animals with genetic modifications of MMP-9 levels. We used MMP-9 KO mice that had no functional MMP-9 and mice with MMP-9 overexpression in the brain. Pentylenetetrazol-induced seizure susceptibility was evaluated in injured animals. The latency between the injection of a subconvulsant dose of PTZ and the first epileptiform discharge confirmed a positive correlation between TBI and seizure susceptibility in the CCI animal model, as previously demonstrated by Bolkvadze and Pitkanen [28]. We also found a strong correlation between MMP-9 genotype and PTZ-induced seizure susceptibility. The latency between the PTZ injection and the first epileptiform discharge was the shortest in MMP-9 OE mice and longest in MMP-9 KO mice.

Continuous vEEG recording for 2 weeks further implicated MMP-9 in the development of PTE after CCI. Approximately 10% of MMP-9 WT mice developed PTE after CCI, and this percentage markedly increased in MMP-9 OE animals (50%) and decreased in MMP-9 KO animals, in which only 6% (1/15) developed seizures. Therefore, we demonstrated a significant functional role for MMP-9 in post-TBI processes that lead to the development of epilepsy. MMP-9 may contribute to epilepsy through mechanisms that involve synaptic plasticity, neuroinflammation, and blood-brain barrier disruption [5, 38, 39].

In the present study, higher MMP-9 levels were associated with a higher prevalence of PTE after brain injury. Moreover, the occurrence of PTE correlated with the lesion area after injury. We suggest that the extent of structural and physiological changes in brain circuitry that occur after injury contribute to epileptogenesis. Notably, structural and functional changes that occur within the dentate gyrus and CA1 field of the hippocampus (mossy fiber sprouting, CA1 degeneration) have been strongly and functionally implicated in different animal models of epilepsy [40–43]. One unresolved issue is whether these changes are related to increases in MMP-9 levels in the hippocampus that are induced by different stimuli, such as TBI. Only 10% of mice developed spontaneous seizures upon TBI, which may be related to the findings in the hippocampus, in which individual differences in MMP-9 levels reflected high heterogeneity in the injured animal group. Importantly, the number of animals that develop spontaneous recurring seizures in this model strongly depends on the strain. Approximately 20% of CD-1 mice develop seizures after TBI [44], whereas only approximately 10% of C57BL/6J mice develop seizures weeks after brain trauma [28].

In a large number of TBI patients, MMP-9 levels increase, which is directly related to physiological inflammatory and repair processes that occur in the injured area and surrounding brain structures. In approximately 20% of TBI patients, MMP-9 levels exceed the threshold of physiological repair, and TBI-induced reorganization becomes excessive, leading to irreversible changes and aberrant rewiring that are the basis of epileptogenesis. Future studies should investigate whether early interventions to lower post-injury MMP-9 levels can prevent pathophysiological processes and reduce the risk of developing epilepsy.
